# Conservation of orbital angular momentum and polarization through biological waveguides

**DOI:** 10.1038/s41598-022-18483-3

**Published:** 2022-08-19

**Authors:** Nicolas Perez, Daryl Preece, Robert Wilson, Anna Bezryadina

**Affiliations:** 1 Department of Physics and Astronomy, California State University Northridge, Northridge, CA 91330 USA; 2 Department of Biomedical Engineering, University of California, Irvine, Irvine, CA 92617 USA; 3 Beckman Laser Institute, University of California, Irvine, Irvine, CA 92697-1475 USA; 4 Department of Medicine, University of California, Irvine, Irvine, CA 92868-3298 USA

**Keywords:** Nonlinear optics, Solitons, Optical manipulation and tweezers, Biophotonics

## Abstract

A major roadblock to the development of photonic sensors is the scattering associated with many biological systems. We show the conservation of photonic states through optically self-arranged biological waveguides, for the first time, which can be implemented to transmit light through scattering media. The conservation of optical properties of light through biological waveguides allows for the transmission of high bandwidth information with low loss through scattering media. Here, we experimentally demonstrate the conservation of polarization state and orbital angular momentum of light through a self-arranged biological waveguide, several centimeters long, in a sheep red blood cell suspension. We utilize nonlinear optical effects to self-trap cells, which form waveguides at 532 nm and 780 nm wavelengths. Moreover, we use the formed waveguide channels to couple and guide probe beams without altering the information. The formed biological waveguides are in a sub-diffusive scattering regime, so the photons’ information degrades insignificantly over several centimeters of propagation through the scattering media. Our results show the potential of biological waveguides as a methodology for the development of novel photonic biosensors, biomedical devices that require optical wireless communication, and the development of new approaches to noninvasive biomedical imaging.

## Introduction

In biological soft-matter environments, such as blood or biological fluids, light typically experiences strong scattering losses, preventing long distance propagation of light. Such losses limit the development of imaging technologies and transmissive light applications^1^, which are essential in the development of deep-tissue imaging, optically controlled biosensors, localized laser treatments, and other medical treatments and diagnosis^2–4^. In recent years, nonlinear optical techniques have been used to overcome strong scattering effects, form optical waveguides, and achieve deep transmission of light through scattering colloidal suspensions^5–12^. The confinement and guiding of light beams have been demonstrated in dielectric, synthetic, metallic, and biological suspensions in which a variety of mechanisms, including: optical polarizability of particles, photophoresis and thermophoresis effects, as well as the particle’s size and shape^6–24^ are instrumental in optical confinement.

Low-loss propagation, nonlinear self-trapping, and the formation of biological waveguides of several centimeters long without significant photodamage to the sample cells has been demonstrated in suspensions of cyanobacteria, E. coli, and red blood cells (RBCs)^12–14^. Since living cells usually have a slightly higher index of refraction than the surrounding media, suspended microorganisms in biological waveguides get attracted toward the center of the continuous-wave (CW) laser beam due to the optical gradient force^5,25–27^ and pushed forward by the forward scattering force^12,13,15^. As a result, hundreds of living cells get trapped along the propagating focused laser beam. The laser beam traps particles near the focus and propels them forward resulting in self-focusing of the beam due to a cumulative particle lensing effect along the beam path, which allows the formation of a biological optical fiber or a biological optical conduit. Furthermore, quite recently, we employed pump/probe-type nonlinear coupling^18,28^ and demonstrated a broad range of wavelengths guided through self-induced waveguide channels formed in RBC suspensions^14^. These achievements clearly show that biological media can exhibit the necessary optical nonlinearity at a large band of wavelengths for transmission deep into scattering media.

In the last decade, several studies have been done on the transmission of various types of complex and structured light dynamics in nanoparticle suspensions^8,9,21–24^. However, unfortunately these observations often cannot be extended to biological suspensions; biological samples are typically comprised of particles larger than nanoparticles (i.e. Mie, not Rayleigh particles), are made from different materials with different polarizability properties, have internal structure, and are sensitive to environmental conditions. Consequently, the mechanism of waveguide formation in biological samples is somewhat different than in solutions of nanoparticles. In biological waveguides, radiation and scattering forces are fundamentally important to the formation of stable waveguides, whereas in nanoparticle waveguides photophoresis and thermophoresis effects play larger roles^21,23^.

In order to use advanced beam shaping techniques for imaging and to facilitate high bandwidth information transmission, it is advantageous to be able to send complex structured light through the highly scattering biological media. Hitherto, this has proven difficult since wavefronts quickly become scrambled in most applications. Beams with orbital angular momentum (OAM or vortex beam) have been used previously in classical and quantum communication^29–32^ and more recently in biomedical applications for noninvasive imaging and diagnosis of tissues^33^. Recent studies indicate possible benefits of using light beams with orbital angular momentum for deep penetration and higher transmittance propagation through dispersive and scattering media^34,35^. This inspired our investigation into the transmission of vortex beam and polarization through biological waveguides.

In this work, we demonstrate experimentally the conservation of polarization and orbital angular momentum through a three centimeter long self-arranged biological waveguide in a sheep RBC suspension for multiple wavelengths. The preservation of orbital angular momentum and polarization are non-obvious especially given the structure of the cells in solution. Furthermore, the propagation dynamics and charge stability of vortex beams in suspensions of live cells are different from that in nanosuspensions. The ability to create waveguides with beams possessing orbital angular momentum and various polarization states, and at a broad spectrum of wavelengths, enables new waveguide structure geometries and expands the bandwidth of information that can be transmitted through the scattering biological media. Therefore, the formed biological waveguide permits transmission of a broad range of signals by varying transmitting beam properties: combination of OAM beams with different levels of topological charge; beams at different wavelengths; beams with a combination of linear and circular polarizations; and time/frequency modulated signals. The ability to send and preserve complex beams yields a better understanding of light and signal propagation in biological fluids and scattering media. Potential applications include transmitting energy and information through scattering media, communication with medical implants, deep-tissue imaging, monitoring internal organs, and improvement of optical biopsy techniques.

## Results

### Light propagation with orbital angular momentum

**Figure 1:**
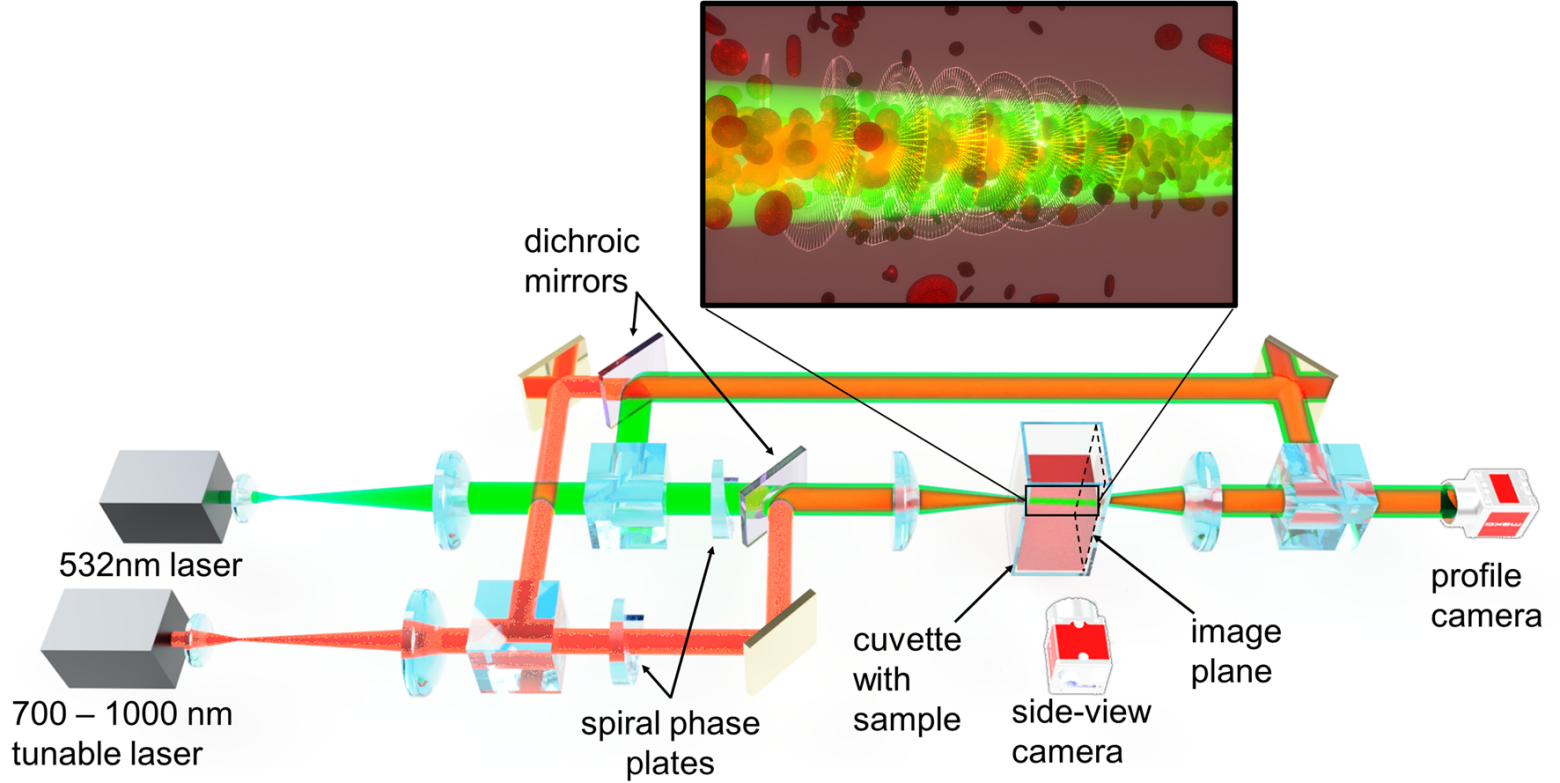
Schematic diagram of the experimental setup. A green and a tunable NIR beam are each sent through spiral phase plates, coaxially combined by a dichroic mirror to propagate collinearly through a sheep RBC suspension. A Mach–Zehnder interferometer path is added to view the phase information of each beam. The effects were recorded using dichroic filters, a beam profile camera, and a side-view camera. (Insert) Illustration of RBCs forming an effective waveguide of light due to the action of optical forces. Cells are attracted toward the center of the beam, and cell concentration decreases radially. OAM modes propagate within the waveguide.

The setup we used to study conservation of orbital angular momentum is sketched in Fig. 1. We modified a pump–probe-type setup^14^ and included a spiral phase plate (topological charge 1) and Mach–Zehnder interferometers for both laser beams (see “Materials and methods” for setup details and sample preparation). The interferometer allows for the detection of orbital angular momentum in the beam after passing through the sample arm of the interferometer by examination of the interference pattern. When a vortex beam and plane wave beam interfere at an angle, a pitch-forked shaped interference pattern is created.

**Figure 2:**
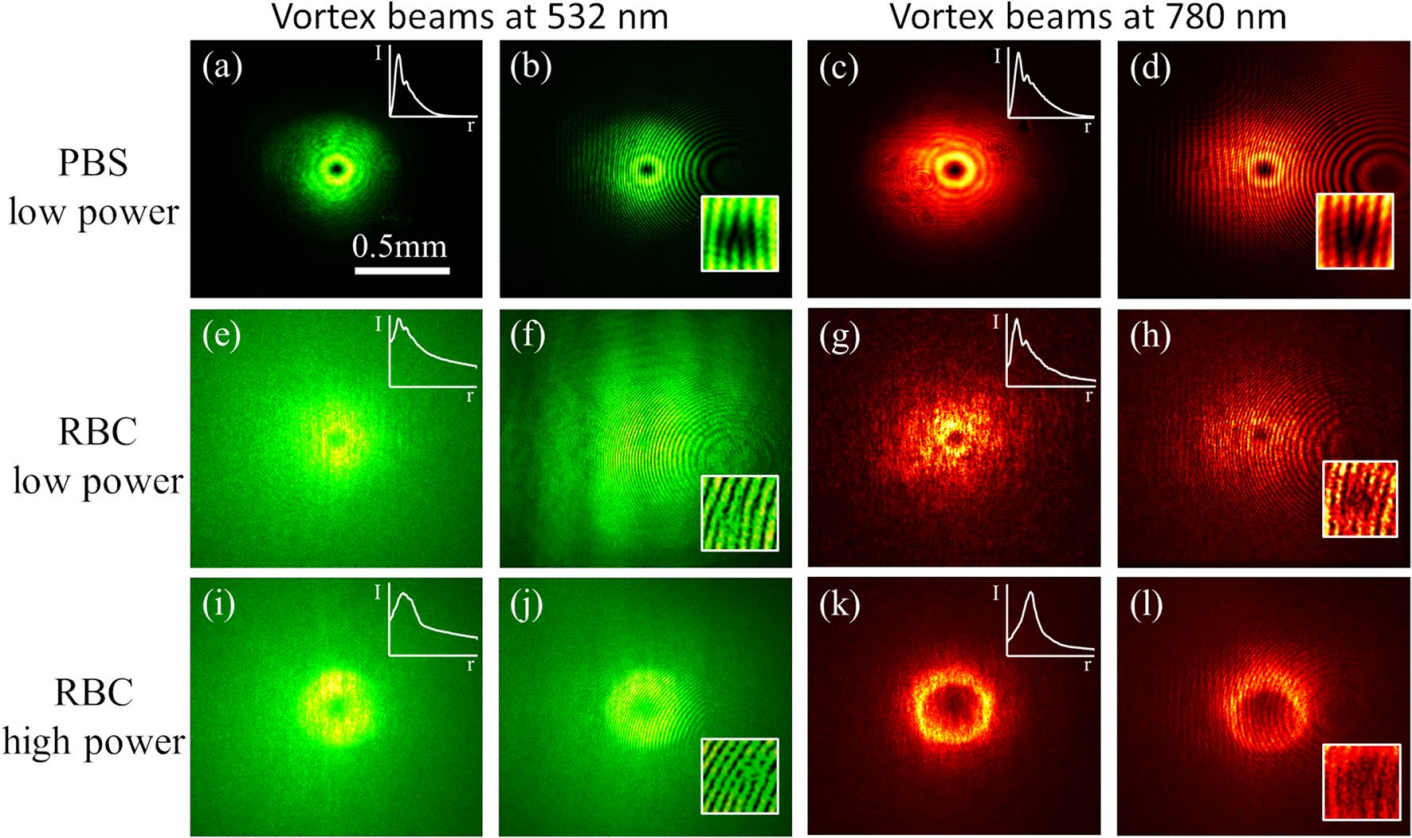
Self-trapping of individual 532 nm green and 780 nm NIR vortex beams through a sheep RBC suspension. (**a**–**d**) Normal diffraction of laser beams through PBS buffer solution without RBCs. (**e**–**h**) Linear diffraction and scattering of the laser beam at low laser power (20 mW) through RBC suspensions. (**i**–**l**) Optimal nonlinear self-trapping and self-focusing of the laser beams at high power. All output beam profiles were imaged at the exit face of the cuvette, (**a**,**c**,**e**,**g**,**i**,**k**) are measurement beams only, (**b**,**d**,**f**,**h**,**j**,**l**) are interferograms between the measurement beam and plane wave reference beam. The inserts in (**a**,**c**,**e**,**g**,**i**,**k**) are the normalized azimuthally averaged radial intensity profiles of the beams.

First, we examine the conservation of optical properties of an individual focused laser beam with orbital angular momentum (vortex beam) through the waveguide. An optical vortex beam has a helical-shaped phase inclination producing a singularity and zero intensity at the center of the vortex^36,37^. In the phosphate buffered saline (PBS) buffer solution without RBCs, the green and NIR vortex beams diffract normally with their singularity preserved at the center (see Fig. 2a–d). In the RBC suspension at low laser powers, scattering causes the vortex beams to expand (Fig. 2e–h). Low power vortex beams do maintain their orbital angular momentum after several centimeters of propagation through the scattering solution, evidenced by the characteristic “pitch-forked” pattern seen on the resulting interferogram (Fig. 2f,h). As the laser power increases to an optimal self-trapping condition (500 mW laser power for 532 nm green laser beam and 1300 mW laser power for 780 nm NIR laser beam), the “donut” intensity pattern is more defined as nonlinear self-focusing reduces the beam’s outer diameter while it expands the inner diameter (Fig. 2i–l). The confinement of light to a reduced beam cross sectional area leads to an increase in the beam’s intensity. Additionally, the overall transmission increases for both beams as they reach the maximum focusing conditions, with an increase in transmission of 5% for the 532 nm beam and 6% for the 780 nm beam through biological scattering media. Self-trapping of an optical beam in the suspension is predominately due to optical gradient and forward-scattering forces^12,13^. Although the possible influence of thermal effects can’t be completely discounted, the thermal and thermophoretic effects will be minimal due to low absorption values at the wavelengths used here^13,14^. RBC suspensions exhibit a wavelength dependent optical nonlinearity, where different laser powers are required for each wavelength to achieve maximum nonlinear focusing and form waveguides^14^. If the laser power increases beyond the optimal self-trapping conditions for a vortex beam, the donut-shaped pattern first becomes thicker and then the beam defocuses due to heating effects.

The light-induced refractive index change in the RBC suspension stabilizes the structured light beam and the donut shaped beam is preserved, propagating several centimeters through the suspension^21^. The vortex waveguide stability is determined by the nonlinearity’s degree of nonlocality; with increased nonlocality, instabilities decrease leading to an increased length of stably propagating waveguide^38^. In our experiments, all vortex beams have a single pitch-forked interference pattern that confirms the presence of a phase singularity after several centimeters of propagation through the RBC suspension (see Fig. 2j,l). A possible explanation for orbital angular momentum preservation can come from the cellular interaction with Laguerre-Gaussian beams, this has previously been shown to cause particles to self-organize into a donut-shaped pattern^39,40^. We might expect a small transfer of orbital angular momentum to the cells^41,42^. However, we do not see evidence of vortex splitting or other signs of change in OAM. It is worth noting that RBCs are very flexible and may aggregate in clumps rather than revolving freely in the waveguide. Thus, the exchange of orbital angular momentum to the cells in our sample could be relatively minute over the 3 cm propagation distance and is undetectable in these experiments.

**Figure 3:**
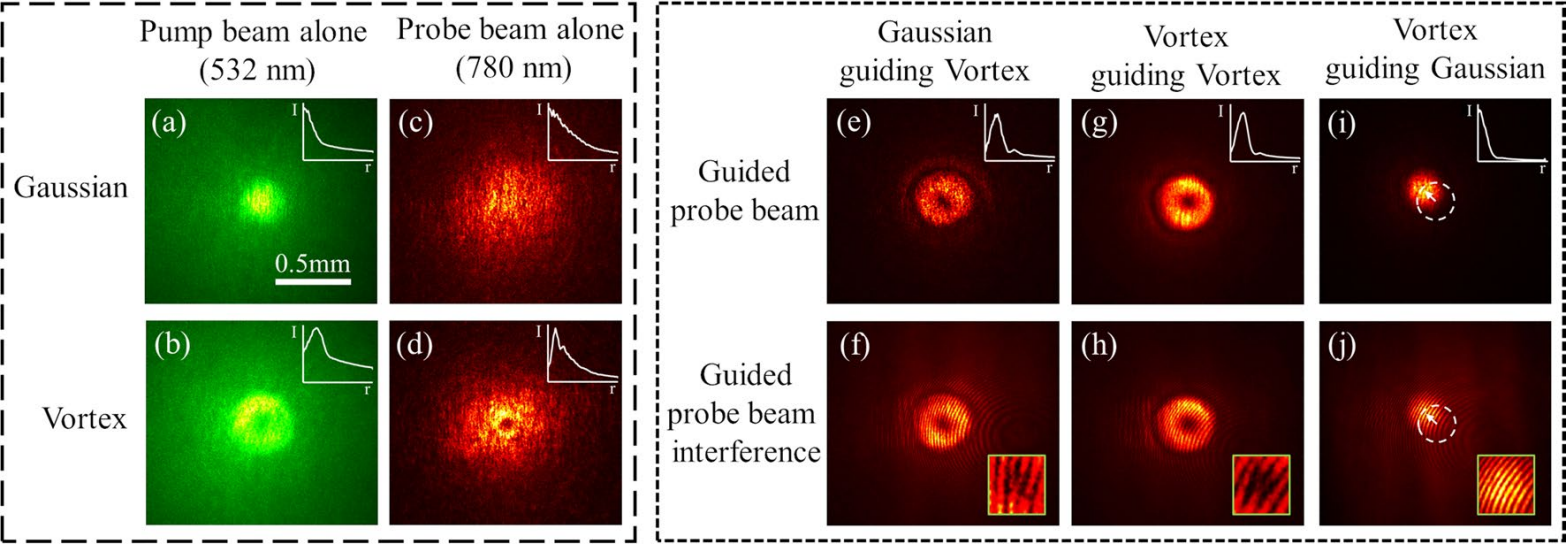
Nonlinear coupling of a low power NIR (780 nm) probe beam by a green (532 nm) pump beam through a sheep RBC suspension. (**a**,**b**) Gaussian and vortex green pump beam profiles at optimal laser power for waveguide formation (500 mW). (**c**,**d**) Gaussian and vortex NIR probe beam profiles at 20 mW laser power. (**e**) Guiding of the vortex probe beam by the green pump Gaussian beam. (**g**) Guiding of the vortex probe beam by the green pump vortex beam. (**i**) Guiding of the Gaussian probe beam by the green pump vortex beam. The probe beam shifts to one side of the vortex beam with the greatest intensity. A dashed circle shows the unguided position of the Gaussian beam. (**f**,**h**,**j**) Interferogram of the guided probe beams (**e**,**g**,**i**), respectively. All images are beam profiles at the exit face of the 3-cm cuvette through the sheep red blood cell sample. The inserts in (**a**,**b**,**c**,**d**,**e**,**g**,**i**) are the normalized azimuthally averaged radial intensity profiles of the beams.

To test for nonlinear coupling between two beams with varying topological charge, we implement a pump-probe-like experiment: a weaker laser beam (probe beam) is guided by a biological waveguide formed by a separate strong beam (pump beam)^14,18^. We use a 532 nm pump beam at 500 mW laser power to create a self-induced waveguide in RBC suspension over a few centimeters, then inject a low power 780 nm probe beam at 20 mW laser power^14,18,23,28^. Both Gaussian (ℓ = 0) and vortex (ℓ = 1) beams are tested as probe and pump beams in various combinations (see Fig. 3a–d).

By itself, the probe beam diffracts linearly without any nonlinear self-action due to its low intensity, as shown in Fig. 3c,d. The probe beam relies on the waveguide created by the pump beam to propagate through the scattering media. When the probe and pump beams are injected simultaneously, vortex probe beams experience the waveguides formed by both Gaussian and vortex pump beams, which result in smaller probe beam output diameters and an increase in the transmission of probe power through the waveguide (Fig. 3e,g). Like with Gaussian pump-probe nonlinear coupling^14^, the strong nonlinear forces from the pump beam produce a high-index waveguide through which the probe beam propagates, confining the probe beam’s power to a small guided region. The guided probe vortex beams maintain their orbital angular momentum properties, as evidenced by the characteristic pitch-forked pattern on the interferogram (Fig. 3f,h). When attempting to guide a Gaussian probe beam through a vortex pump beam waveguide, the Gaussian probe beam tends to veer off to the side of the “donut” with the greatest intensity (Fig. 3i,j). This suggests the distribution of cells that produce the high index region is tubular for a vortex pump beam, with a high index region along the donut-shaped beam and low index regions outside and along the singularity at the vortex center. Both vortex and Gaussian beams are capable of nonlinear coupling into waveguide channels and both preserve their intensity distribution and orbital angular momentum proprieties. With this evidence, we conclude that a waveguide formed by either Gaussian or vortex light is able to guide both Gaussian and vortex beams at different wavelengths at lower power.

### Conservation of polarization through RBC waveguides

In our next experiments, we investigate the conservation and transmission of polarization states of both pump and probe beams through scattering RBC suspensions. To create a linearly or circularly polarized beam, we modify the pump-probe-type setup and include a polarizer, a flipping half-wave plate, and a flipping quarter-wave plate before the laser beams are focused into the RBC suspension (see “Materials and methods” and Fig. S1). The output polarization states are detected by introducing a polarizer (analyzer), a quarter-wave plate, and a power detector. This configuration and the removable flipping elements, enables the creation of consistent vertically/horizontally linearly polarized and right/left circularly polarized beams and detection of any possible alternation of polarization state after propagating through the suspension.

**Figure 4:**
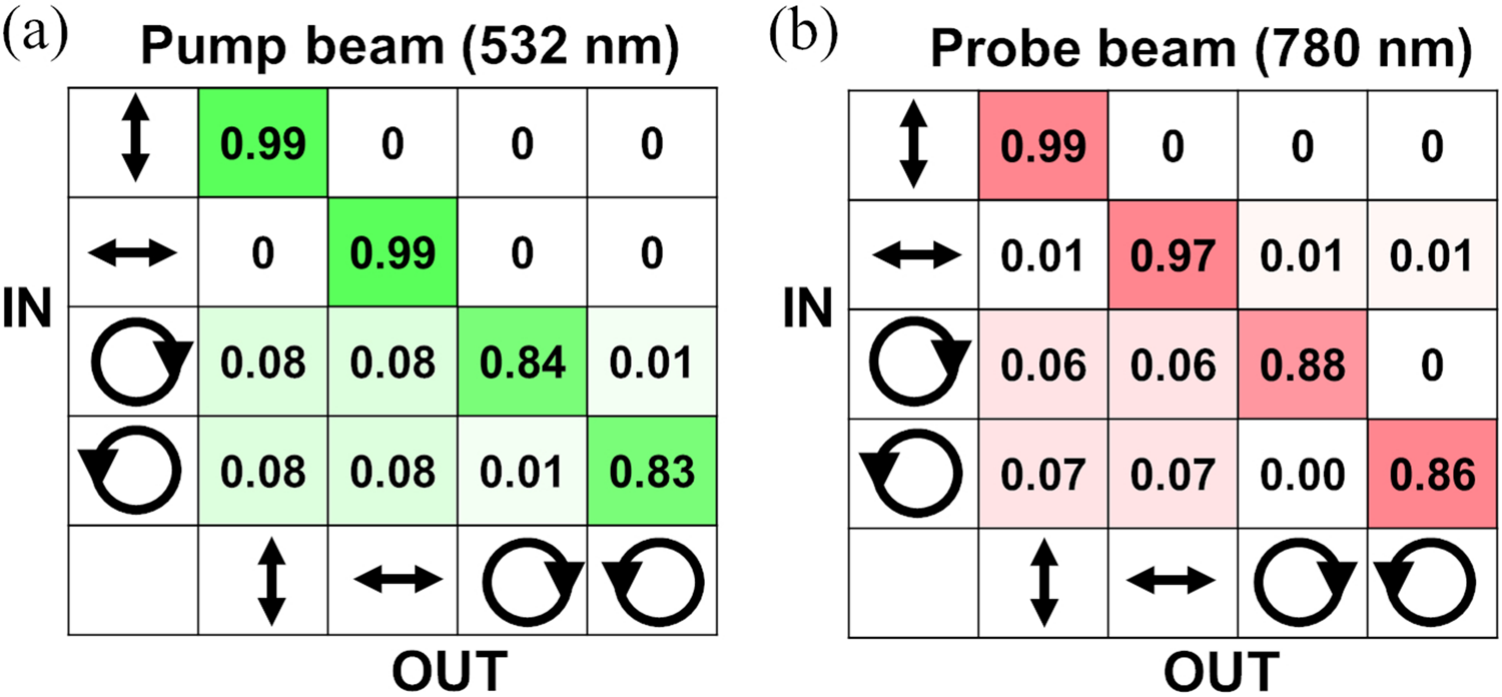
The output intensity distribution between polarization states (vertical, horizontal, right circular, and left circular) after propagating through the RBC suspension for different input polarization states of the laser beam. (**a**) The intensity distribution for the pump beam alone (532 nm) at 500 mW. (**b**) The intensity distribution for the probe beam (780 nm) with guiding. The uncertainty for linearly polarized light is 0.01 and for circular polarized light is 0.09.

Figure 4 illustrates the output intensity distribution between polarization states for the pump beam alone (532 nm) at 500 mW laser power and for the guided probe beam (780 nm) at 20 mW laser power. To detect any changes in intensity distribution for each polarization state, we measure output transmission as a function of the analyzer angle, normalize the measured values to the powers transmitted through the PBS background media to account for the difference in optical components, fit a sinusoidal curve to the data points, and determine any shifts in phase and amplitude of sinusoidal function. See supplementary information S2 for additional details and plots of sine waves for each polarization state. We found no principal difference in the propagation and behavior of the pump beams (at high laser power, which is needed to observe the nonlinear process) and the probe beams (at low laser power). All four polarization states are preserved after propagating through the 3-cm cuvette with RBC suspension, as shown in Fig. 4. However, the transmission power slightly varies depending on polarization. Circular polarization states have a large error due to the strong sensitivity of two quarter-wave plates alignment, a slight difference in retardance of achromatic waveplates, and a small fluctuation of laser power. The linearly polarized beam has fewer optical components in its path and is less sensitive to alignment, so the uncertainty in the polarization measurements is several times smaller than for circular polarized light. Our results are consistent with colloidal suspensions of metallic nanoparticles, for which beams maintain their intensity profile and polarization structure throughout the nonlinear transformation at high laser power^21^. The structural and polarization stability of probe beams in biological suspensions allows for the transmission of broadband signal thought waveguides formed by a pump beam.

## Discussion

In this work, we demonstrated the successful conservation of the orbital angular momentum and polarization state of a beam sent through a suspension of sheep RBCs several centimeters in length. The nonlinearity and the formation of biological waveguides do not alter the transmitting information. It is a significant result that the ring-shaped beams are stable in propagation in the nonlinear regime through biological scattering media. The observed mechanism of waveguide formation in biological suspensions is different from metallic nanoparticle suspensions, where thermo-optical forces play a crucial role in beams prorogation and stability^21^. Due to gradient and forward-scattering forces, cells are attracted toward the beam, providing a local change of the refractive index of the suspension which forms a waveguide. Once formed by one beam, the waveguide can support the stable propagation of beams with different wavelength, polarization, angular momentum, and intensity distribution, which are crucial parameters for the precise control of light-matter interaction. In the future, we are interested in studying the propagation of probe beams with a wide range of sizes and with large OAM. For a more rigorous analysis of the OAM mode purity after propagating through biological suspensions, a spatial OAM mode-decomposition can be performed^43^ based on collected beam profiles and interference images or, alternatively, an OAM mode sorter^44^ can be used to measure mode purity experimentally. With the “ring” technique^45^, which was used to study optical vortices in fiber, it is also possible to characterize the mode purity of structured beams with the superposition of multiple OAM modes combined with polarization states via spin-orbital interaction.

**Figure 5:**
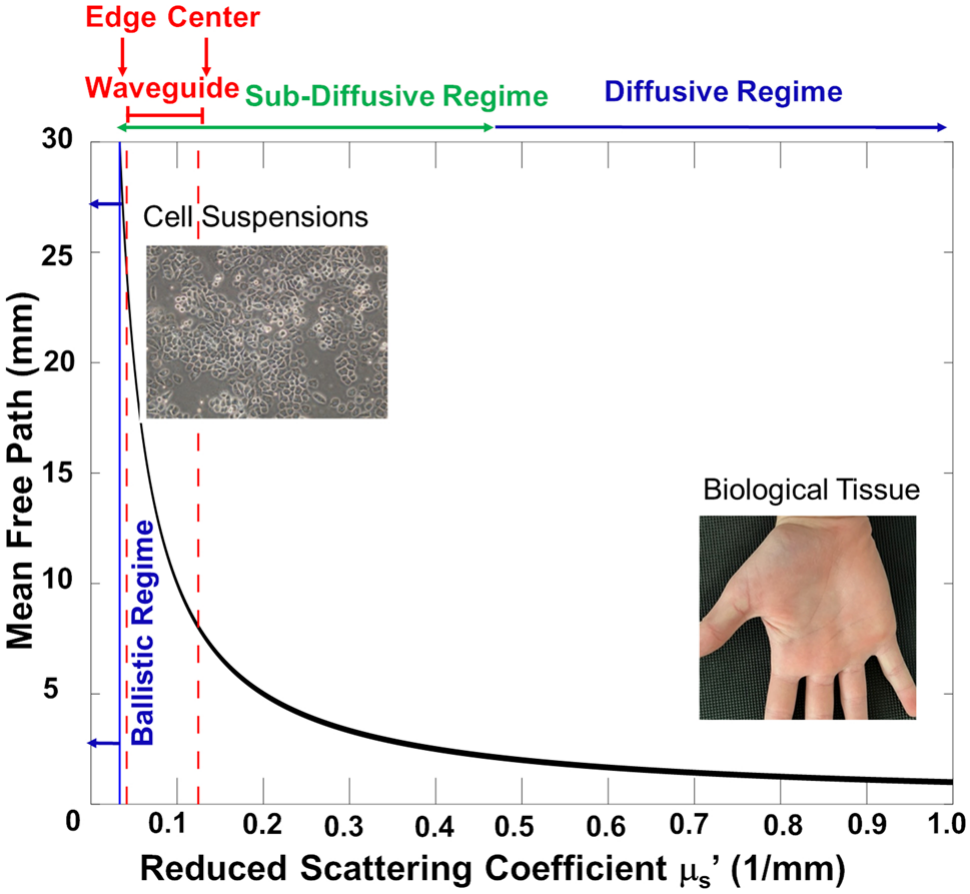
Ballistic, sub-diffusive, and diffusive scattering regimes for photons incident on a 3 cm length enclosure containing scattering media. The mean free path of a photon in the medium is equal to the reciprocal of the reduced scattering coefficient *μ*_*s*_*ʹ*. The center and edge of the formed waveguide in the suspension of red blood cells are within the sub-diffusive light scattering regime, wherein minimal information loss due to degradation of polarization and coherence is expected. For reduced scattering coefficients less than 1/30 = 0.033 mm^−1^, the mean free path becomes larger than the length of the container (30 mm), so photon transport can be approximated as ballistic. As *μ*_*s*_*ʹ* increases sufficiently to allow more than ~ 10 scattering events within the container (*μ*_*s*_*ʹ*  ~ 0.4–0.5 mm^−1^), the transport of light begins to approach the diffusive regime (as is the case with biological tissue, which typically has *μ*_*s*_*ʹ*  ~ 0.8–1.5 mm^−1^), wherein a sufficient number of scattering events occur that the initial polarization and coherence states of the light are degraded.

As light propagates through the suspensions, multiple scatting events can occur. For strongly scattering media, we would expect a substantial mode splitting for orbital angular momentum due to momentum transfer. However, for our biological suspensions, we observe only slight degradation in polarization and undetectable by regular interferometer mode splitting for OAM. To further understand and characterize the capability of the waveguide to preserve the polarization and orbital angular momentum states of the incident light, we modeled the reduced scattering coefficient within the distribution of cells using Mie scattering theory^46^ (see “Materials and methods”). The Mie theory model, along with the approximation that the scattering anisotropy (mean cosine of scattering angle) of the cells is roughly 0.9^47^, provided an estimate of the reduced scattering coefficient *μ*_*s*_*ʹ* ~ 0.04 mm^−1^ for the suspension. However, since the concentration of the cells is approximately 3 times higher in the middle than toward the edges of the waveguide^12^, we estimate that *μ*_*s*_*ʹ* ranges from 0.04 to 0.12 mm^−1^ over the distribution of RBCs within the waveguide (see Fig. 5). To understand the effect of this scattering coefficient on the degradation of the information of the input beam state, it is crucial to know the number of scattering events that a typical photon from the beam would undergo due to the cells in the waveguide. The mean free path (MFP) of a photon in the medium can be obtained as follows:

1 $$MFP=1/({\mu }_{s}^{^{\prime}}+{\mu }_{a})\approx 1/{\mu }_{s}^{^{\prime}}$$

where *μ*_*a*_ is the absorption coefficient of the medium, which can be considered negligible relative to the reduced scattering coefficient for this experimental setup. For our suspension, the mean free path is MFP ~ (1/0.04) = 24 mm at the edges of the waveguide and MFP ~ (1/0.12) = 8 mm at the center of the waveguide. Previous literature on diffuse light transport reports that photon trajectories start to become randomized after 1–2 mean free paths^48^ and that the number of photons for which polarization information is preserved degrades by a factor of 1 − (1/*e*) ~ 63% following 6 scattering events^49^. Since the length of the cuvette in our study was 3 cm long and 1 MFP ~ 2.4 cm near the edges of the waveguide, it is reasonable to assume that photon transport toward the edges of the waveguide is nearly ballistic. Furthermore, since 1 MFP ~ 0.8 cm toward the center of the waveguide, we approximate that only 3–4 scattering events occur for photons near the center of the waveguide. Therefore, even at the center of the waveguide, the light paths can be treated as “sub-diffuse” and a significant amount of polarization information should be preserved. As Fig. 5 illustrates, the scattering regime throughout the biological waveguide in the suspension is far from that of a highly diffusive turbid material such as biological tissue. The light scattering within the biological waveguide does not produce enough scattering events to degrade the information significantly from the initial state of the light.

For this study, the scattering coefficients of the medium were estimated by using a Mie calculator^50^, using estimated scatter concentrations at different locations along the waveguide^12^. In future studies, the scattering and absorption coefficients of the suspension can be measured directly by using an integrating sphere technique^51^. It may also be possible to directly quantify the scattering profile across the waveguide during the experiment by detecting the backscattered light with the side-view camera and calibrating this signal against the backscattered signal from a tissue-simulating material with known absorption and scattering properties similar to that of the suspension^52^.

The current propagation losses for waveguides in RBC suspensions are high compared to standard fiber optic materials and are mainly attributable to the scattering in colloidal suspensions. Overall transmission decreases with distance, and the power transmission is typically up to 8% for visible and NIR light after 3 cm of propagation distance through our concentration of RBC in suspensions. The formed waveguide helps to focus the transmitted laser power in the narrow channel of light, which aids in delivering signals to a target-specific area. Additionally, only a small portion of light is absorbed by the cells^13,14^; and laser damage is minimal in RBC suspensions, as previous studies have shown^13^. We expect that future improvement in finding optimal materials for biological suspensions will lead to more favorable propagation losses.

In conclusion, our observations confirm that a large bandwidth of information can be guided through biological scattering media with minimum altering and loss of information. The demonstrated structural stability of complex beams in biological suspensions opens up potential possibilities to implement structured light to transmit light and information deep through otherwise highly scattering bio-soft-matter, communicate with medical implants, improve optical biopsy techniques and imaging, and to fabricate biological optical circuits and biosensors^3^. Furthermore, the transmitted structured light can be used for various applications: microscopy^1^, Raman spectroscopy^53^, optical spectroscopy^2^, particle trapping and manipulation^54^, and second harmonic generation^55^. This work thoroughly demonstrates that the formed biological waveguides are good choices for transmission of a broad range of signals toward the small target area in biological fluids and scattering media.

## Materials and methods

### Sample preparation

We dilute 2 µl of pure RBC’s (Innovative Research, ISHRBC100P15ML) in 8.5 ml phosphate buffered saline 7.4 pH (PBS) which results in an optimal cell concentration to form optical waveguides while maximizing transmission. To ensure a consistent cell concentration, we use a spectrophotometer to calibrate the sample to an absorption of 0.203 cm^−1^ at 532 nm wavelength with reference to a PBS background. The sheep RBC has a biconcave disk shape with a mean cell diameter of 4.3 µm and a thickness of 1.4 µm.

### Experimental setup for light propagation with orbital angular momentum

The positions of optical elements in the setup are shown in Fig. 1. The experimental setup includes two continuous-wave linearly polarized laser beams: a green 532 nm laser (Msquared, Equinox DPSS pump, λ = 532 nm) and a NIR 780 nm laser (Msquared, SolsTiS 4000 PSX XF, tunable Ti–Sapphire, λ = 700–1000 nm). 532 nm and 780 nm wavelengths were selected for their strong self-focusing nonlinearity and increased transmission in RBC suspensions^14^. Both beams are independently collimated, coaxially aligned using dichroic mirrors, and focused by a 100 mm lens 0.8 cm inside of a 3-cm long glass cuvette filled with a sheep RBC suspension. The full width half maximum (FWHM) focus spot sizes of the beams are 17 µm for the green laser and 18 µm for the NIR laser (for λ = 780 nm). Topological charge 1 spiral phase plates (RCP Photonics, VPP-1c and Vortex Photonics, V-532-10-1) are added for each laser, just before the two beams are combined with a dichroic mirror. To observe phase information of the beams individually, a Mach–Zehnder interferometer is added for both laser beams. A reference portion of each collimated, plane wave beam is redirected by a beam splitter and recombined with the sample beam by another beam splitter after the sample to examine the interference at the imaging plane. A CCD beam profile camera (Newport, SP928) records the interference patterns and the beam’s input/output profiles through the sample.

### Experimental setup for light propagation with different polarization states

The modified pump-probe-type experimental setup includes a polarizer, a flipping superachromatic half-wave plate, and a flipping superachromatic quarter-wave plate before the beams are focused inside of the 3-cm long cuvette containing the RBC suspension, as illustrated in Fig. S1. This enables the creation of vertically/horizontally linearly polarized and right/left circularly polarized beams. The output polarization state is detected by using a polarizer (analyzer) and a power detector after the beam passes through an imaging lens and a filter. To analyze circularly polarized light, a flipping achromatic quarter-wave plate is inserted to change the circularly polarized beam back into a linearly polarized beam, which allows us to detect any possible alternation of polarization state after propagating through the RBC suspension. All wave plates were locked in their mounts to maintain a consistent polarization state for the duration of the experiments.

### Modeling of the reduced scattering coefficient using mie scattering theory

To calculate the scattering coefficient of the sheep RBC suspension in PBS media, we used a Mie calculator^50^ with the following input parameters: mean cell diameter = 4.3 μm, cell concentration by volume = 0.023%, cell refractive index = 1.4, medium refractive index = 1.335. The output scattering coefficient *μ*_*s*_ was estimated to be 0.4 mm^−1^ for a homogeneous distribution of red blood cells in the medium. Based on a previous theoretical modeling of particles distribution inside of biological waveguide^12^, the concentration of cells toward the middle of the waveguide is expected to be approximately 3 times the concentration toward the edges. Therefore, based on this Mie theory model, *μ*_*s*_ ranges from ~ 0.4 to 1.2 mm^−1^ from the edges to the center of the waveguide. The reduced scattering coefficient *μ*_*s*_*ʹ* can be obtained by the equation:

2 $$\mu^{\prime}_{s} = \mu_{s} (1 - g)$$

where *g* is the anisotropy of the medium (mean cosine of scattering angle). For a sample of red blood cells *g* is approximately 0.9^47^. Therefore*, μ*_*s*_*ʹ* is expected to range from 0.04 to 0.012 mm^−1^ over the distribution of red blood cells within the waveguide.

## Supplementary Information

Supplementary Information.

## Data Availability

The datasets used and/or analyzed during the current study available from the corresponding author on reasonable request.
