# Supplementary material for: Conservation of orbital angular momentum and polarization through biological waveguides
Source: Sci Rep. 2022 Aug 19;12:14144. doi: 10.1038/s41598-022-18483-3 (PMC9391334; doi:10.1038/s41598-022-18483-3)
Supplement: Supplementary file 1 — Supplementary Information. [file 41598_2022_18483_MOESM1_ESM.docx]

**Supplementary Information:**

**Conservation of orbital angular momentum and polarization through biological waveguides**

Nicolas Perez^1,2,3^, Daryl Preece^2,3^, Robert Wilson^3,4^, Anna Bezryadina^1*^

*^1^ Department of Physics and Astronomy, California State University Northridge, Northridge, CA, 91330, USA*

*^2^ Department of Biomedical Engineering, University of California, Irvine, Irvine, CA, 92617, USA*

*^3^ Beckman Laser Institute, University of California, Irvine, Irvine, CA, 92697-1475, USA*

*^4^ Department of Medicine, University of California, Irvine, Irvine, CA, 92868-3298, USA*

Corresponding author: A. Bezryadina, Email: [anna.bezryadina@csun.edu](mailto:author_three@uni-jena.de)

**S1 Experimental setup to determine polarization states**

The following figure illustrates the experimental setup to investigate the polarization states and transmission power of individually propagating 532 nm and 780 nm self-trapped Gaussian beams and a guided low power NIR probe beam by a green pump beam.


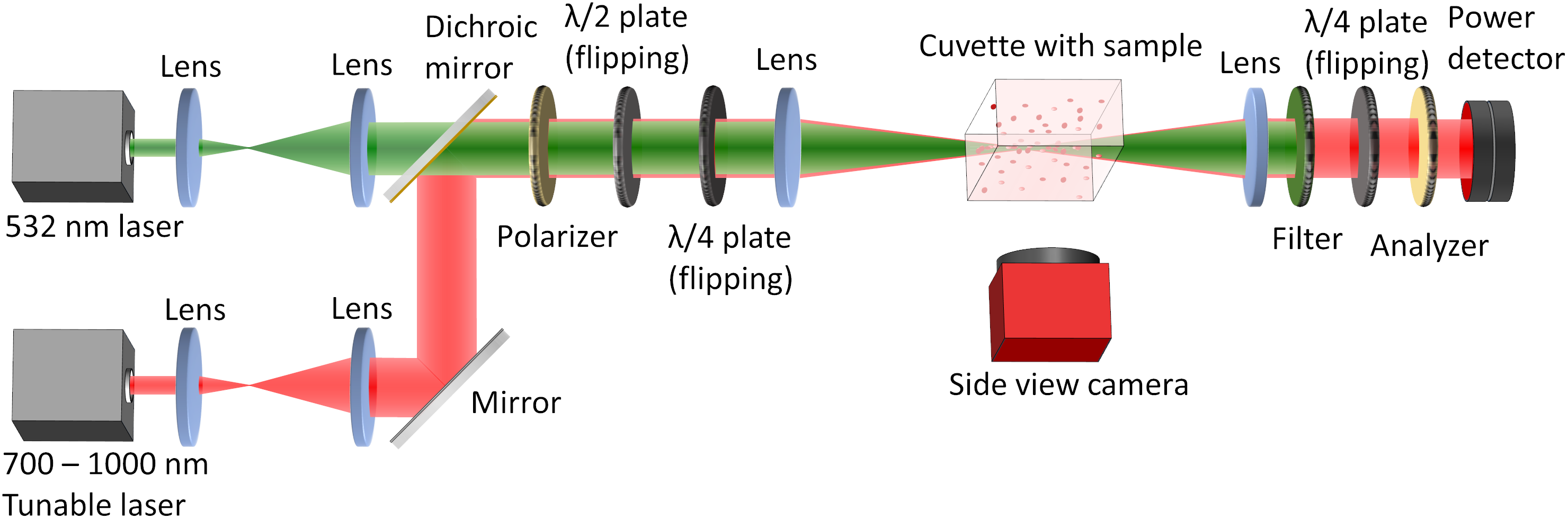


**Figure S1.** **Schematic diagram of the experimental setup to test conservation of polarization states through RBC suspensions.** To produce linear or circular polarized light, half-wave and quarter-wave plates are included in the path of the green beam and the tunable NIR beam. Both beams are combined, propagating collinearly through a sample with RBC suspension. The beam output polarization state is detected by an analyzer, a flipping quarter wave plate, and a power detector.

**S2 Transmission power of light for different polarization states**

Figure S2 illustrates the normalized transmission power of light for different polarization states; vertical or horizontal linear polarization and right or left circular polarization. To detect any changes in polarization state after propagating through the suspension, the transmitted power is measured for different angles of the polarizer (analyzer). The power measurements are normalized to the powers transmitted through the PBS background media to take into account the different optical components in the optical path of each beam. The polarization graphs show a small shift in phase between polarization measurements in PBS media and RBC suspensions. The polarization graphs also maintain a sinusoidal shape where each state is distinctly identifiable after transmission through the scattering solution. This implies that all four polarization states are preserved after propagating through the 3-cm cuvette. However, the transmission power slightly varies depending on polarization. In our experiments, the circular polarization graphs do not reach zero due to a slightly misaligned quarter-wave plate. We observe maximum transmission values of 3.6% for the 532 nm self-guiding beam, 7.9% for the 780 nm self-guiding beam, and 7.3% for the 780 nm probe beam guided by the 532 nm pump beam. These transmission values are consistent with previous data for guiding at multiple wavelengths.^1,2^ Finally, we did not observe any significant difference in polarization transmission for guided and unguided low power probe beams (Figure S2c,d).


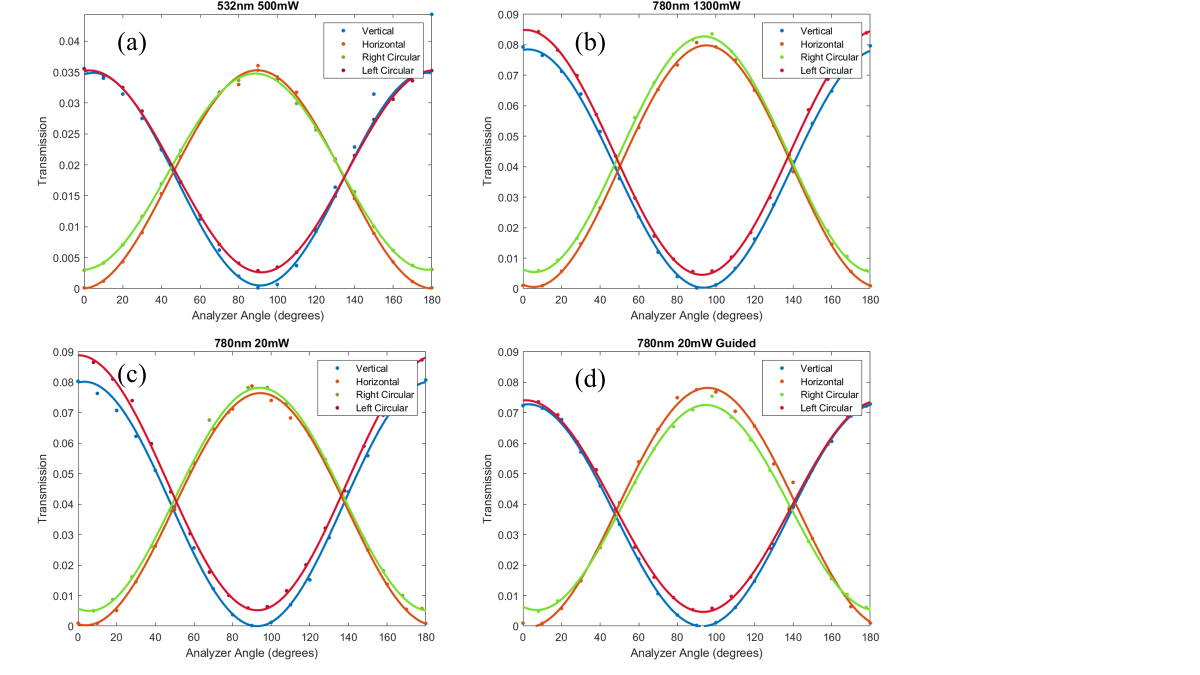


**Figure S2.** **Graphs of analyzed polarization transmission of beams through RBC media with vertical (blue), horizontal (orange), right circular (green), and left circular (red) polarization states.** **(a)** Polarization states of the pump beam alone (532 nm) at 500 mW. **(b)** Polarization states of the pump NIR beam alone (780 nm) at 1300 mW. **(c)** Polarization states of the weak probe beam (780 nm) at 20 mW with no guiding. **(d)** Polarization states of the weak probe beam (780 nm) with guiding. Transmission values are taken as the ratio of analyzed guided light and maximum transmission in a pure PBS media. Overlapping linear and circular states are easily differentiated experimentally by the presence of a quarter-wave plate.

**References**

1. Perez, N., Chambers, J., Chen, Z., & Bezryadina, A. Nonlinear self-trapping and guiding of light at different wavelengths with sheep blood. *Opt. Lett.* **46(3)**, 629 (2021).
2. Kelly, T. S., *et al.* Guiding and nonlinear coupling of light in plasmonic nanosuspensions. *Opt. Lett.* **41**, 3817 (2016).
